# Non-retroviral Endogenous Viral Elements in Tephritid Fruit Flies Reveal Former Viral Infections Not Related to Known Circulating Viruses

**DOI:** 10.1007/s00248-023-02310-x

**Published:** 2023-12-01

**Authors:** Luis Hernández-Pelegrín, Vera I. D. Ros, Salvador Herrero, Cristina M. Crava

**Affiliations:** 1 Department of Genetics and University Institute of Biotechnology and Biomedicine (BIOTECMED), Universitat de València, Dr Moliner 50, 46100 Burjassot (Valencia), Spain; 2 Laboratory of Virology, Wageningen University and Research, Droevendaalsesteeg 1, 6708 PB Wageningen, The Netherlands

**Keywords:** Evolutionary virology, Paleovirology, Insect-virus ecology, Insect immunity, Small RNA

## Abstract

A wide variety of insect-specific non-retroviral RNA viruses specifically infect insects. During viral infection, fragments of viral sequences can integrate into the host genomes creating non-retroviral endogenous viral elements (nrEVEs). Although the exact function of nrEVEs is so far unknown, some studies suggest that nrEVEs may interfere with virus replication by producing PIWI-interacting RNAs (piRNAs) that recognize and degrade viral RNAs through sequence complementarity. In this article, we identified the nrEVEs repertoire of ten species within the dipteran family Tephritidae (true fruit flies), which are considered a major threat to agriculture worldwide. Our results suggest that each of these species contains nrEVEs, although in limited numbers, and that nrEVE integration may have occurred both before and after speciation. Furthermore, the majority of nrEVEs originated from viruses with negative single-stranded RNA genomes and represent structural viral functions. Notably, these nrEVEs exhibit low similarity to currently known circulating viruses. To explore the potential role of nrEVEs, we investigated their transcription pattern and the production of piRNAs in different tissues of *Ceratitis capitata.* We successfully identified piRNAs that are complementary to the sequence of one nrEVE in *C. capitata,* thereby highlighting a potential link between nrEVEs and the piRNA pathway. Overall, our results provide valuable insights into the comparative landscape of nrEVEs in true fruit flies, contributing to the understanding of the intimate relation between fruit flies and their past and present viral pathogens.

**Supplementary Information:**

The online version contains supplementary material available at 10.1007/s00248-023-02310-x.

## Introduction

Tephritid fruit flies pose a major threat to agriculture worldwide. Around ten per cent of the 4000 species composing the Tephritidae family are considered a pest, including members of the genera *Anastrepha, Bactrocera, Ceratitis, Rhagoletis* and *Zeugodacus* [1]. To control their spread in agricultural areas, the sterile insect technique (SIT) has been the most extensively employed method and has successfully reduced crop production losses [2–5]. SIT involves the production and systematic area-wide release of sterile males. However, a significant concern in the mass production of males and their competitive ability is the presence of insect pathogens. These pathogens can lead to outbreaks in reared colonies, or can produce covert infections that have detrimental effect on the released males [6].

Insect specific viruses (ISVs) specifically infect insect hosts, mainly causing covert infections with negligible behavioural and physiological effects [7]. However, in some cases, they can result in overt infections that lead to the death of the insects [8]. Over the past decade, the increasing availability of high throughput RNA sequencing data has reshaped our understanding of the RNA virome in a range of species across kingdoms. This wealth of data has also unveiled numerous ISVs that infect true fruit fly species. In particular, two RNA viruses have been identified in *Zeugodacus* species, 36 in seven *Bactrocera* species and 13 in the Mediterranean fruit fly, *Ceratitis capitata* [9–12] (Table S1). These viruses can represent a threat to mass rearing facilities, but, more importantly, they can influence their host’s ecology by affecting fitness and physiology, as well the immune status [13, 14].

In insects, the cornerstone of viral immunity is represented by the small interfering RNA (siRNA) pathway [15]. However, in *Aedes* mosquitos, another class of small RNAs called PIWI-interacting RNAs (piRNAs) has been discovered to interfere with RNA virus replication [16–18]. This phenomenon has not been observed in *D. melanogaster*, where the piRNA pathway does not have clear antiviral role [19]. The extent of this mechanism in other dipteran species, such as true fruit flies, remains unknown. The piRNAs function by recognizing and degrading RNAs through nucleotide complementarity. The first described and primary role of piRNAs is to repress transposable elements (TEs) in germline cells [20, 21]. Within this system, some genomic island called piRNA clusters are enriched in TE sequences and undergo modulation following the mobilization of novel TEs, thereby serving as a heritable immune memory against TEs [20]. However, it has been recently described that during viral infection, fragments of viral sequences can integrate into the insect genomes creating endogenous viral elements (EVEs). The presence of EVEs has been reported for different insect genomes [22–25] and in *Aedes* mosquitoes, some of these EVEs produce piRNAs that reduce virus replication [18]. Viral integration is a process necessary to complete the replication of retroviruses [26]. However, integration of fragments from viral genomes can also occur for non-retroviral RNA viruses, which lack an RNA-dependant RNA polymerase in their genomes and do not go through a DNA stage during their cycle [22, 24, 25]. This step may be facilitated by the activity of a host RNA-dependent RNA polymerase [17]. If viral integration happens in the germline, the resulting non-retroviral EVE (nrEVE) will be vertically transmitted to the progeny remaining in the genome as a marker of past infections that occurred even millions of years ago [27].

This study focuses on the identification of nrEVEs within the large dipteran family of Tephritidae. Specifically, we analysed ten true fruit fly species that are of high economic interest. These species were selected as a comparative framework to gain insights into their nrEVE landscapes. Our results show that each of these species contains nrEVEs ranging from 1 to 8 in most species, except for *Eutreta diana* containing 22 nrEVEs. The majority of these nrEVEs derive from viruses of the *Rhabdoviridae* family, originate from viral regions that encoded for structural proteins and show low sequence similarity with the genomes of currently known circulating RNA viruses. Using RNA-seq data, we further analysed the transcriptional patterns of nrEVEs in a selected number of species to identify potential transcripts. Lastly, we investigated whether *C. capitata* nrEVEs produce piRNAs, to unravel the potential antiviral role of nrEVEs in these species.

## Methods

### Viral Protein Database and Tephritid Fruit Fly Genomes

To identify non-retroviral endogenous viral elements (nrEVEs) in the genomes of tephritid fruit flies, a viral database was created including 4787 protein sequences from viruses classified within the *Riboviria* realm, and infecting invertebrates (NCBI Virus, accessed in September 2021). In addition, protein sequences from RNA viruses recently described in arthropods were included in the database to increase the chances of finding divergent viral sequences [10, 28–30].

Tephritid fruit fly species were selected based on the availability of a reference genome, and their relevance as current or potential agricultural pest. Following these criteria, we investigated the presence of nrEVEs in the reference genomes of *Ceratitis capitata*, *Eutreta diana*, *Ragholetis zephyria*, *Tephritis californica, Trupanea jonesi,* and five species of *Bactrocera: Bactrocera (Zeugodacus) cucurbitae, Bactrocera dorsalis, Bactrocera latifrons, Bactrocera oleae,* and *Bactrocera tryoni.* Accession numbers and quality parameters of the reference genomes are displayed in Table S2.

### Characterization of nrEVEs Repertoire

For the nrEVEs identification, each reference genome was annotated separately using a blastx search against the above-mentioned viral protein database. The E-value cut-off for blastx identification was set to 10^-6^, with the rest of the settings left as default. To verify the viral origin of the putative nrEVEs, each sequence was mapped back to the NCBI non redundant (nr) protein database using blastx search with an E-value cut-off of 10^-4^ [24]. nrEVEs sequences with high similarity to host insect proteins, retroviruses or transposable elements were discarded. For those putative nrEVEs mapping to viral sequences, the top viral hit was considered for taxonomic classification. nrEVEs names include a four letters abbreviation of the host name, the root of the viral family originating the nrEVE, and a number to differentiate the nrEVEs with the same viral origin present in each fruit fly species.

### Nucleotide Similarity Between nrEVEs and Circulating Viruses

Blastn was used to identify the similarities between nrEVEs and RNA viruses infecting tephritid fruit flies at the nucleotide level. Fifty genomes of tephritid fruit fly RNA viruses were recovered from the NCBI nucleotide repository and recent publications [9–12] to create the database for the analysis (Table S1). The cut-off for blastn identification was set to 50% query cover and 50% nucleotide identities, with the remaining algorithm parameters maintained as default.

### nrEVEs Distribution Along Viral Genomes

To identify whether some viral genomic regions are more prone to generate nrEVEs, nrEVEs were individually aligned to the genome of a reference virus, which was specific for each viral family. Blastx against the nr protein sequence database filtered by viruses (taxid: 10239) was employed for the identification of the reference viral genome, and ClustalW (v2.0) for the multiple alignment. The results of the alignments were visualized using BioEdit (Hall, T.A. et al., 1999) to assess the start and end positions of the nrEVEs. The distribution analysis was only performed for those viral families represented by more than ten nrEVEs: *Partitiviridae* and *Rhabdoviridae*.

### Assessment of nrEVEs Transcription

First, the availability of RNA-seq data in the Sequence Read Archive (SRA) was determined for each of the tephritid fruit fly species. Four different RNA-seq experiments were selected for each of the six species with available datasets. Selection was based on sequencing technology (Illumina) and library construction strategy (paired-end reads). When possible, samples from different developmental stages (eggs, larvae, pupae, adult) were included. SRA accession numbers and characteristics are listed in the Table S3. The quality of the sequenced reads was analysed using fastQC (https://www.bioinformatics.babraham.ac.uk/projects/fastqc/). Raw datasets were then trimmed with Trimmomatic [31] to remove adaptors and low-quality sequences. The remaining reads were de novo assembled using Trinity-v2.9.0 [32]. The presence of nrEVEs in the assembled contigs was determined using blastn with default parameters and an E-value cut-off of 10^-4^.

Secondly, the 53 *C. capitata* RNA-seq datasets available at NCBI were investigated in silico for the presence of nrEVEs (Table S4). SRA reads were trimmed with Trimmomatic [31] and mapped against the nrEVEs described in *C. capitata* reference genome using Bowtie 2 v 2.3.5.1 [33]. nrEVEs transcription levels were determined using RSEM v 1.3.1 [34] with default parameters. The relative abundance of each nrEVE was calculated relative to the endogenous L23a gene of *C. capitata* and represented using the heatmap.2 function from the gplots v 3.1.1 package in R [35].

### Small RNA Sequencing

Small RNAs were sequenced from ovaries and somatic tissues from two *C. capitata* strains (Control and Wild-F4). The control strain was established in 2001 using wild flies captured at experimental fields located in Moncada (Valencia, Spain), and has been reared under laboratory conditions for more than 100 generations. The Wild-F4 strain (W) derives from *C. capitata* pupae collected on infested figs fruits (*Ficus carica*) from commercial citrus orchards located in Alcira (Valencia, Spain) in August 2020. Differently from the control strain, Wild-F4 strain has been reared in laboratory conditions for only four generations. In both cases, laboratory conditions were 26 °C, 40–60 % humidity, and 14/10h light/dark cycles (Arouri et al., 2015).

Virgin adult females from each population were isolated after emergence and reared separately from the adult males with water and food provided *ad libitum*. Eight days after emergence virgin females were collected and dissected using entomological tweezers and pins to isolate the ovaries whereas the thorax and the head were used as somatic tissues. Immediately after dissection, the samples were preserved in RNA later (Sigma Aldrich R091, St. Louis, MO, USA). RNA extraction was performed with the TriPure isolation reagent (cat. No. 11667157001; Roche, Mannheim, Germany) using pools of ten heads/thoraxes and 20 ovaries. RNA integrity and quantity were measured using 1% agarose gel electrophoresis and spectrophotometry.

Small RNA library construction and sequencing were performed by Macrogen (Seoul, Korea). Libraries were obtained using the TruSeq Small RNA Library Prep Kit (Illumina, San Diego, CA, USA) and sequencing was run in the HiSeqX platform (Illumina). Paired end reads of 150 nt and 1 Gb of raw data were generated from each of the four libraries under analysis (NCBI; SAMN21882268, SAMN21882269, SAMN21882270, and SAMN21882271).

### Characterization of nrEVE-Derived Small RNAs

Small RNA-Seq reads were bioinformatically treated to eliminate adapters, empty sequences, low complexity sequences, and reads with more than 20% low-quality [36]. After cleaning, reads were filtered by length using custom Perl scripts, and only the sequences between 18 and 32 nt were maintained. nrEVEs-derived sRNA sequences were identified by mapping the clean reads against the sequences of the nrEVEs described in medfly using Bowtie 2 v 2.3.5.1 [33]. Identical reads were merged to calculate the percentage of nrEVE-derived sRNAs from each sRNA library mapping to each nrEVE. The mapping position of the sRNA reads along the nrEVEs was visualized using an Integrative Genomics Viewer [37] and the length distribution, base composition, and strand distribution of the sRNAs were analysed using a custom Python script described by Lewis et al., 2018 (accessible on GitHub: https://github.com/SamuelHLewis/sRNAplot) [38].

## Results

### The Genomes of Tephritid Fruit Flies Harbor nrEVEs

**Fig. 1:**
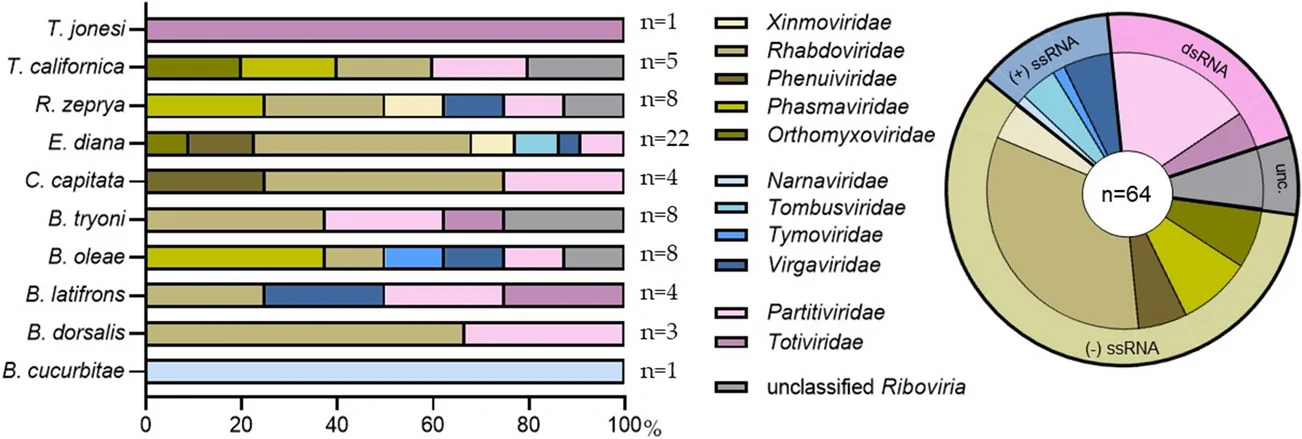
Classification of nrEVEs from the reference genomes of ten tephritid fruit flies. The nrEVEs were assigned to a viral family according to the top hit of blastx searches against the non-redundant database on NCBI. Viral families are shown using different colours, as specified in the legend. **A** Bar graph shows the percentage of nrEVEs assigned to each viral family for each tephritid fruit fly. The total number (n) of nrEVEs detected in each tephritid fruit fly species is shown on the right side of the bar graph. **B** The pie chart shows the proportion of the nrEVEs described in tephritid fruit flies which derive from each viral family. The total number of nrEVEs (n) is indicated

**Fig. 2:**
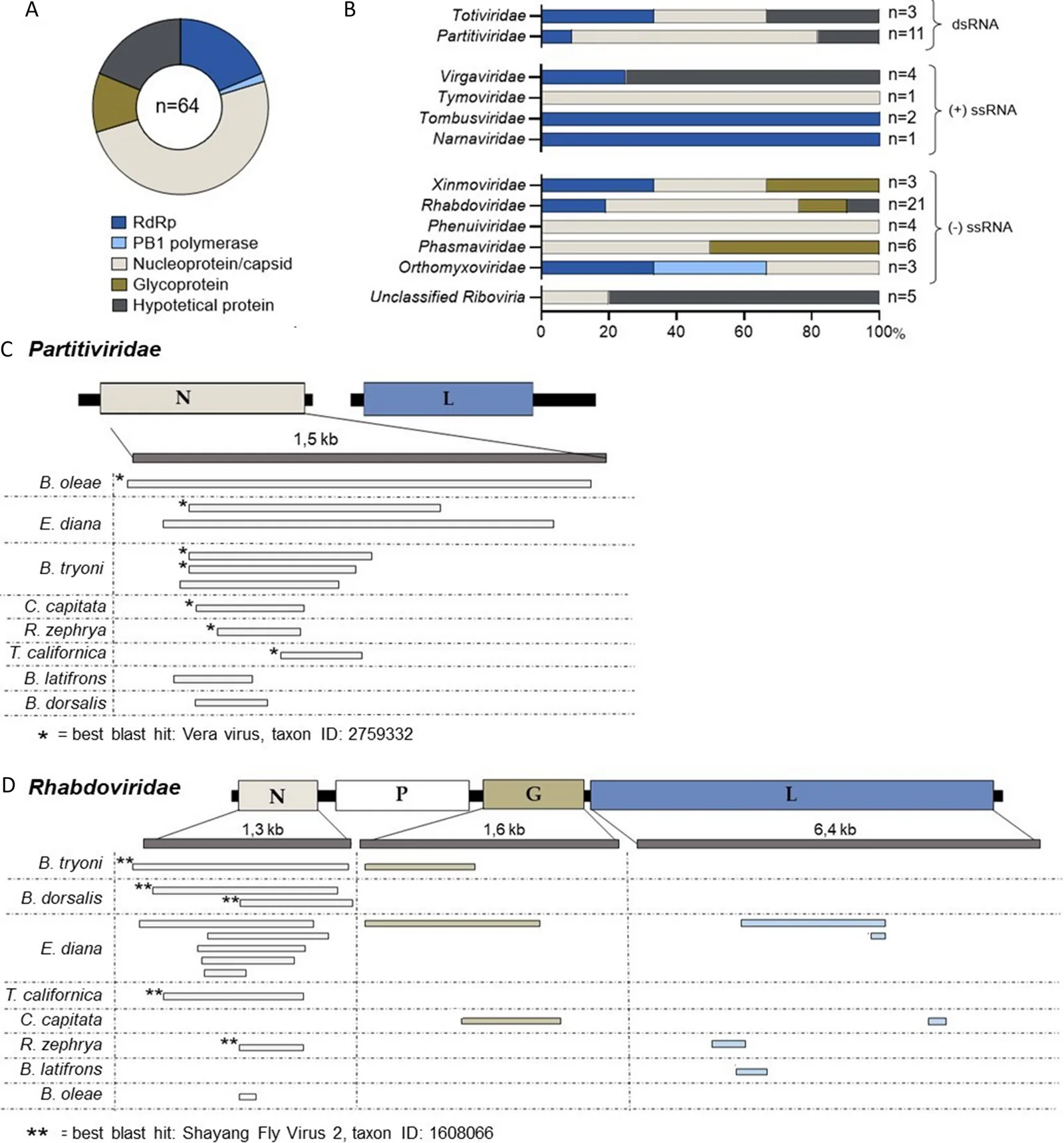
Functions encode in the viral regions originating the nrEVEs. **A** Pie chart of the viral functions assigned to the tephritid nrEVEs. **B** Distribution of the viral functions within each viral family. The total number of nrEVEs per viral family is displayed on the right of the bar graph. **C** and **D** nrEVEs distribution across a representative viral genome. The genomic structure of a representative member of *Partitiviridae* (**C**) and *Rhabdoviridae* (**D**) is shown in the upper panel. Boxes represent the viral ORFs, and viral functions are indicated in capital letters: N (capsid), G (Glycoprotein), L (RNA-dependent RNA polymerase), P (uncharacterized). Best blastx hits shared by different nrEVEs are shown. nrEVEs are displayed according to their length and mapping position on the representative viral genome

A total of 64 nrEVEs were identified and distributed among the species as follows: *Bactrocera (Zeugodacus) cucurbitae* (*n=*1), *Bactrocera dorsalis* (*n=*3), *Bactrocera latifrons* (*n=*4), *Bactrocera oleae* (*n=*8), *Bactrocera tryoni* (*n=*8), *Ceratitis capitata* (*n=*4), *Eutreta diana* (*n=*22), *Rhagoletis zephyria* (*n=*8), *Tephritis californica* (*n=*5), and *Trupanea jonesi* (*n=*1) (Fig. 1). The number of annotated nrEVEs varied among the selected genomes, with *E. diana* having the highest number of nrEVEs (*n=*22), while some species had a single nrEVE (*T. jonesi* and *B. cucurbitae*) (Fig. 1). In some cases, nrEVEs were found to cluster together within the same genomic region (Table 1). For example, two chromosomes of *B. oleae* contained nrEVEs that were less than 5000 bp apart. Similarly, two scaffolds of *R. zephrya* contained two nrEVEs located within 1000 bp of each other. In the case of *E. diana,* three scaffolds contained nrEVEs that were less than 200 bp apart, with *EVE_EdiaRhabdo_9* and *EVE_EdiaRhabdo_10* being separated by only 60 bp. Despite their proximity, we considered these nrEVEs to be distinct entities based on their distribution across the sequence of their representative virus (Fig. 2D). Moreover, due to the high fragmentation of some reference genomes, it is possible that the nrEVEs clusters are more numerous than reported here.

**Table 1:** Characterization of the 64 nrEVEs retrieved in the genomes of tephritid fruit flies

nrEVE name	Tephritid fruit fly	Accession^a^	nt start^b^	nrEVE length	Viral region^c^	Transcript^d^
nrEVE_BcucNarna_1	*B. cucurbitae*	NW_011863697.1	79633	221	RdRp	0/4
nrEVE_BdorPartiti_1	*B. dorsalis*	NW_011869496.1	975	215	Capsid	3/4
nrEVE_BdorRhabdo_2	*B. dorsalis*	NW_011874984.1	8863	1175	Capsid	3/4
nrEVE_BdorRhabdo_1	*B. dorsalis*	NW_011876088.1	199702	719	Capsid	3/4
nrEVE_BlatToti_1	*B. latifrons*	NW_017534628.1	40607	134	RdRp	0/4
nrEVE_BlatVirga_1	*B. latifrons*	NW_017534907.1	8651	149	Hypothetical	1/4
nrEVE_BlatPartiti_1	*B. latifrons*	NW_017536152.1	2791	233	Hypothetical	0/4
nrEVE_BlatRhabdo_1	*B. latifrons*	NW_017537113.1	49913	485	RdRp	4/4
nrEVE_BoleUnc_1	*B. oleae*	LGAM02010267.1	1012	281	Hypothetical	0/4
nrEVE_BolePartiti_1	*B. oleae*	LGAM02013578.1	63099	1376	Capsid	0/4
nrEVE_BoleTymo_1	*B. oleae*	LGAM02022319.1	8889	419	Capsid	0/4
nrEVE_BoleVirga_1	*B. oleae*	LGAM02022319.1	9879	173	Hypothetical	0/4
nrEVE_BoleRhabdo_1	*B. oleae*	LGAM02022611.1	270159	107	Capsid	4/4
nrEVE_BolePhasma_3	*B. oleae*	LGAM02022787.1	16056	773	Glycoprotein	0/4
nrEVE_BolePhasma_2	*B. oleae*	LGAM02022787.1	20198	862	Glycoprotein	0/4
nrEVE_BolePhasma_1	*B. oleae*	LGAM02022787.1	24720	1228	Glycoprotein	0/4
nrEVE_BtryUnc_2	*B. tryoni*	JHQJ01000026.1	128451	125	Hypothetical	0/4
nrEVE_BtryRhabdo_1	*B. tryoni*	JHQJ01000078.1	417965	1373	Capsid	1/4
nrEVE_BtryPartiti_3	*B. tryoni*	JHQJ01001339.1	64465	473	Capsid	0/4
nrEVE_BtryUnc_1	*B. tryoni*	JHQJ01002975.1	7854	449	Hypothetical	0/4
nrEVE_BtryRhabdo_2	*B. tryoni*	JHQJ01003828.1	34340	701	Glycoprotein	0/4
nrEVE_BtryPartiti_1	*B. tryoni*	JHQJ01008948.1	8943	542	Capsid	0/4
nrEVE_BtryToti_1	*B. tryoni*	JHQJ01010486.1	2654	254	Hypothetical	0/4
nrEVE_BtryPartiti_2	*B. tryoni*	JHQJ01022891.1	142	494	Capsid	0/4
nrEVE_CcapPhenui_1	*C. capitata*	NW_019376369.1	394469	290	Capsid	3/4
nrEVE_CcapRhabdo_1	*C. capitata*	NW_019378575.1	201736	629	Glycoprotein	4/4
nrEVE_CcapRhabdo_2	*C. capitata*	NW_019378578.1	708556	276	RdRp	0/4
nrEVE_CcapPartiti_1	*C. capitata*	NW_019378583.1	1219447	320	Capsid	4/4
nrEVE_EdiaRhabdo_7	*E. diana*	JXPB01001061.1	1811	769	Capsid	-
nrEVE_EdiaTombus_2	*E. diana*	JXPB01011939.1	2277	851	RdRp	-
nrEVE_EdiaRhabdo_6	*E. diana*	JXPB01023432.1	2431	680	Capsid	-
nrEVE_EdiaRhabdo_2	*E. diana*	JXPB01041133.1	515	266	Capsid	-
nrEVE_EdiaRhabdo_5	*E. diana*	JXPB01041133.1	867	590	Capsid	-
nrEVE_EdiaRhabdo_4	*E. diana*	JXPB01048060.1	1429	347	Hypothetical	-
nrEVE_EdiaPhenui_3	*E. diana*	JXPB01052776.1	249	751	Capsid	-
nrEVE_EdiaOrthomyxo_2	*E. diana*	JXPB01059388.1	811	1281	Polymerase PBI	-
nrEVE_EdiaRhabdo_8	*E. diana*	JXPB01060825.1	762	1104	Capsid	-
nrEVE_EdiaRhabdo_3	*E. diana*	JXPB01062097.1	1703	335	Hypothetical	-
nrEVE_EdiaParititi_1	*E. diana*	JXPB01069909.1	442	746	Capsid	-
nrEVE_EdiaVirga_1	*E. diana*	JXPB01074003.1	450	227	RdRp	-
nrEVE_EdiaRhabdo_9	*E. diana*	JXPB01076293.1	482	1115	Glycoprotein	-
nrEVE_EdiaRhabdo_10	*E. diana*	JXPB01076293.1	1657	2305	RdRp	-
nrEVE_EdiaTombus_1	*E. diana*	JXPB01078316.1	975	682	RdRp	-
nrEVE_EdiaXinmo_2	*E. diana*	JXPB01081550.1	1865	1308	RdRp	-
nrEVE_EdiaRhabdo_1	*E. diana*	JXPB01083515.1	1285	224	Capsid	-
nrEVE_EdiaXinmo_1	*E. diana*	JXPB01092275.1	1377	1193	Glycoprotein	-
nrEVE_EdiaPhenui_1	*E. diana*	JXPB01133418.1	21	248	Capsid	-
nrEVE_EdiaPhenui_2	*E. diana*	JXPB01133418.1	461	263	Capsid	-
nrEVE_EdiaOrthomyxo_1	*E. diana*	JXPB01138291.1	668	668	Capsid	-
nrEVE_EdiaPartiti_2	*E. diana*	JXPB01138318.1	85	1213	Hypothetical	-
nrEVE_RzepPartiti_1	*R. zephrya*	NW_016157090.1	263471	248	Capsid	-
nrEVE_RzepRhabdo_2	*R. zephrya*	NW_016157090.1	274927	521	RdRp	-
nrEVE_RzepuncRib_1	*R. zephrya*	NW_016157156.1	16917	470	Capsid	-
nrEVE_RzepVirga_1	*R. zephrya*	NW_016157156.1	17688	179	Hypothetical	-
nrEVE_RzepPhasma_1	*R. zephrya*	NW_016157315.1	176535	281	Capsid	-
nrEVE_RzepPhasma_2	*R. zephrya*	NW_016157416.1	457	500	Capsid	-
nrEVE_RzepRhabdo_1	*R. zephrya*	NW_016203273.1	307	408	Capsid	-
nrEVE_RzepXinmo_1	*R. zephrya*	NW_016204950.1	724	650	Capsid	-
nrEVE_TcalPartiti_1	*T. californica*	JXPN01014811.1	1303	241	Capsid	-
nrEVE_TcalRhabdo_1	*T. californica*	JXPN01043797.1	48	890	Capsid	-
nrEVE_TcalOrthomyxo_1	*T. californica*	JXPN01049893.1	3	224	Capsid	-
nrEVE_TcaluncRib_1	*T. californica*	JXPN01093879.1	9	713	Hypothetical	-
nrEVE_TcalPhasma_1	*T. californica*	JXPN01108971.1	252	848	Capsid	-
nrEVE_TjonToti_1	*T. jonesi*	JXQA01001198.1	1315	221	Capsid	-

^a^Accession to the genomic region of the host in which the nrEVE was identified

^b^Start position of the nrEVE in the genomic region of the host in which it was identified

^c^Function associated to the viral region originating the nrEVE

^d^Frequency of detection of the nrEVEs in randomly selected transcriptome datasets (*n* = 4 per host species) of tephritid fruit flies

### The nrEVEs Have Diverse Viral Origins and Are Not Related to Circulating Viruses

The 64 nrEVEs identified in tephritid fruit flies derived from 11 different viral families, excluding five nrEVEs which were unclassifiable beyond the *Riboviria* realm (*n=*5; 8%) (Fig. 1). The majority of nrEVEs derived from negative ssRNA viruses (*n=*37; 58%) from the following families: *Rhabdoviridae* (*n=*21), *Phasmaviridae* (*n=*6), *Orthomyxoviridae* (*n=*3), *Phenuiviridae* (*n=*4), and *Xinmoviridae* (*n=*3) (Fig. 1). Among these, the *Rhabdoviridae*–derived sequences were the most abundant (*n=*21; 33%), and they were identified in eight out of the ten tephritid fruit fly species (Fig. 1). On the other hand, nrEVEs derived from positive ssRNA viruses were less common (*n=*8; 13%), and they were distributed across four families: *Virgaviridae* (*n=*4), *Tombusviridae* (*n=*2), *Narnaviridae* (*n=*1), and *Tymoviridae* (*n=*1) (Fig. 1). Finally, dsRNA viruses contributed to a total of 14 nrEVEs (22%), originating from two different families: *Partitiviridae* (*n=*11) and *Totiviridae* (*n=*3). Similar to *Rhabdoviridae-*derived nrEVEs, albeit in lesser abundance, *Partitiviridae*-derived nrEVEs were identified in eight out of ten tephritid fruit fly species (Fig. 1).

When comparing the nrEVEs with the 51 RNA viruses infecting tephritid fruit flies and described so far in the existing literature (Table S1), we found that only five nrEVEs exhibited certain similarity at the nucleotide level with currently known circulating viruses. However, the percentage of nucleotide identity in all cases was lower than 80% (Table 2). Furthermore, we observed that none of the tephritid species that hosted the queried nrEVEs matched with the known hosts of the best matching circulating virus obtained through blastn, except for *nrEVE_CcapRhabdo_1* and its host Ceratitis capitata sigmavirus (CcaSV) infecting *C. capitata*. Nevertheless, the nucleotide similarity between them was only 66%.

**Table 2:** Nucleotide sequence similarity between nrEVEs and circulating viruses. Only results with more than 50% query cover (QC) and nucleotide identities (Identity) are included

Name	Chromosome	Length	blastn (nucleotide vs nucleotide)			
			QC (nrEVE)	e-value	Identity	Circulating viruses
nrEVE_BcucNarna_1	NW_011863697.1	221	72%	2E-34	78.88%	Ceratitis capitata narnavirus
nrEVE_BlatRhabdo_1	NW_017537113.1	485	86%	2E-101	79.19%	Ceratitis capitata sigmavirus
			74%	9E-49	72.33%	Bactrocera tryoni rhabdovirus 1
			71%	5E-40	70.49%	Bactrocera dorsalis sigmavirus
nrEVE_BtryRhabdo_2	JHQJ01003828.1	701	66%	3E-25	65.89%	Ceratitis capitata sigmavirus
nrEVE_CcapRhabdo_1	NW_019378575.1	629	60%	5E-22	66.24%	Ceratitis capitata sigmavirus
nrEVE_EdiaXinmo_2	JXPB01081550.1	1308	89%	2E-120	67.85%	Bactrocera dorsalis xinmovirus 2

Most of the nrEVEs found in the genomes of tephritid fruit flies originated from viral regions that encoded structural viral proteins (*n=*39; 61%), specifically the capsid protein (*n=*32; 50%) and the glycoprotein (*n=*7; 11%). nrEVEs derived from genes encoding non-structural proteins were three times less abundant (*n=*14; 22%), and they were mainly derived from the viral gene encoding the RNA-dependent RNA polymerase (*n=*13; 20%) (Table 1 and Fig. 2A).

Several nrEVEs distributed across the genomes of various fruit flies exhibited overlapping viral open reading frame (ORF) and shared the same best blastx hit (Fig. 2). For instance, seven *Partitiviridae*-derived nrEVEs distributed across five species had the Vera Virus as best blast hit and originated from the ORF that encodes the nucleoprotein (Fig. 2C). Similarly, five overlapping *Rhabdoviridae*-derived nrEVEs generated from the ORF encoding the nucleoprotein had the Shayang fly virus 2 as best blast hit and were present in *B. tryoni*, *B. dorsalis*, *T. californica* and *R. zephyra* (Fig. 2C). These findings raise the possibility that these overlapping nrEVEs are orthologues originated from a viral insertion that occurred in the ancestor of the existing lineages. However, it is important to note that the nucleotide identity of the overlapping regions is relatively limited (Figure S1). On the other hand, the majority of nrEVEs we identified in our study did neither shared the same best blastx hit nor originated from the same the viral region. This suggests that they are independent insertions that occurred in the existing lineages.

Additionally, we identified potentially paralog nrEVEs that may have originated by duplication events that occurred within the host. For example, in the genome of *B. oleae*, we retrieved three nrEVEs located in the same contig and derived from the transcript encoding the glycoprotein of *Phasmaviridae*. Of them, the longest *nrEVE_Bole_Phasma_1* shared close to 100% homology at the nucleotide level with *nrEVE_Bole_Phasma_2* in its 5’region, and with *nrEVE_Bole_Phasma_3* in the 3’region (Figure S1).

### Some nrEVEs Can Be Transcribed and Processed Through the piRNA Pathway

To assess the transcriptional activity of nrEVEs, we explored the presence of transcripts derived from the nrEVEs in publicly available transcriptomic datasets. Six out of the ten species under analysis had multiple public datasets: *B. cucurbitae, B. dorsalis, B. latifrons, B. oleae, B. tryoni* and *C. capitata* (for a total of 28 nrEVEs). From these, we randomly selected four datasets per species (Table S3). Our results revealed that ten out of the 28 nrEVEs analysed were actively transcribed (Table 1). They derived from *Rhabdoviridae* (*n=*6), *Partitiviridae* (*n=*2)*, Phenuiviridae* (*n=*1) and *Virgaviridae* (*n=*1) families, and mainly represented structural viral proteins as the capsid protein (7/10) and the glycoprotein (1/10).

To determine whether the transcribed nrEVEs could encode for viral proteins, we investigated the presence of open reading frames (ORFs) within their sequences. Among the transcribed nrEVEs, *nrEVE_CcapRhabdo_1*, *nrEVE_BdorRhabdo_1* and *nrEVE_BdorRhabdo_2* contained ORFs of 117, 255 and 305 amino acids in length, respectively (Table 1). According to blastx alignments, these amino acidic sequences represent only a partial fragment of structural viral proteins.

Next, we focused on the four nrEVEs identified in *C. capitata*, a world-wide distributed and devastating agricultural pest with ample genomic resources available. First, the presence of the four medfly nrEVEs was confirmed in ten genomic datasets originating from six diverse medfly strains distributed worldwide (Table S5). To expand our analysis of transcriptional patterns, we included data from 53 medfly transcriptomes obtained from NCBI (Table S4). The results confirmed that *nrEVE_CcapRhabdo_1* and nr*EVE_CcapPartiti_1* were consistently transcribed. In contrast, *nrEVE_CcapPhenui_1* transcription varied between samples (36/53), and transcripts derived from *nrEVE_CcapRhabdo_2* were barely detected (7/53) (Figure S2).

**Fig. 3:**
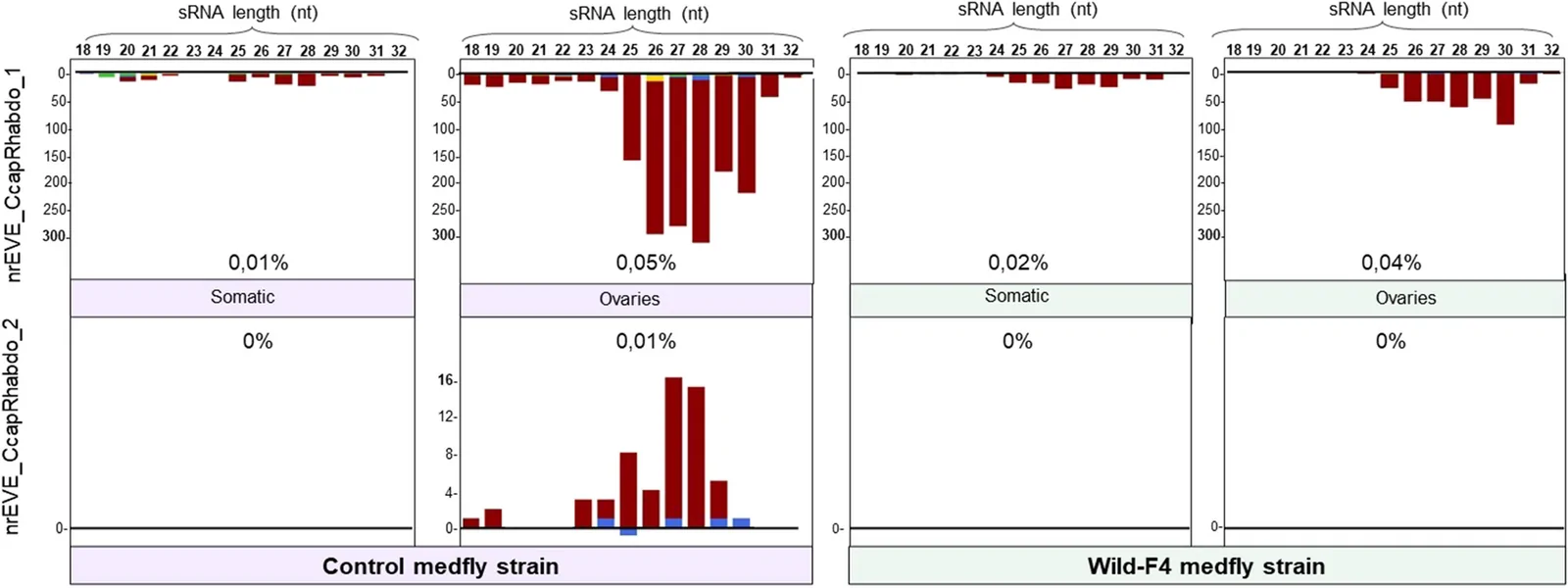
sRNA profile of *nrEVE_CcapRhabdo_1* and *nrEVE_CcapRhabdo_2* in somatic tissues and ovaries of the control and wild-F4 medfly strains. sRNA reads were filtered by length (18 to 32 nt). Positive strand reads are shown above the solid horizontal line while negative-strand reads are shown below. The sRNA counts are shown in the vertical axis. Percentages indicate the number of unique reads mapping to each EVE sequence compared to the total unique sRNA reads obtained for each sRNA library. Colours indicate the first nucleotide of the sRNA read (red, U; green, A; blue, C; yellow, G)

Given that the production of piRNAs by nrEVEs in mosquito ovaries has been suggested to play a protective role against viral infections and decrease transovaric transmission of viruses [18], we aimed to determine whether *C. capitata* nrEVEs generate piRNAs. For this purpose, we sequenced small RNAs from ovaries and somatic tissues of two *C. capitat*a strains (Control and Wild-F4). The results revealed that a fraction of the total sRNAs (< 0.5% of total sRNAs) mapped to two out of the four nrEVEs, namely *nrEVE_CcapRhabdo_1 and nrEVE_CcapRhabdo_2* (Fig. 3). The mapped sRNAs exhibited a distinctive peak between 25 and 30 nt in length and were produced by only one of the strands of the nrEVE. Additionally, nrEVE-derived sRNAs had a bias of uracil as first nucleotide (Fig. 3), which is a common characteristic of piRNAs. Altogether, these observations strongly support the notion that the sRNAs mapping to the nrEVEs were indeed piRNAs.

piRNAs produced by *nrEVE_CcapRhabdo_1* were detected in both *C. capitata* strains, with higher abundance in ovaries than somatic tissues, especially in the control strain. In this strain, the ovaries were also responsible for the production of few piRNAs that mapped against *nrEVE_CcapRhabdo_2*, which were not detected in Wild-F4 strain (Fig. 3).

We then mapped the in silico identified piRNAs back to the nrEVEs from which they originated, and no specific hotspots were found (Figure S3). Moreover, we mapped the piRNAs originated from *nrEVE_CcapRhabdo_1* against Ceratitis capitata sigmavirus (CcaSV), which is the most similar known circulating virus to the nrEVE. Only three piRNAs exhibited sequence similarity to CcaSV, although it was limited to 22 or 24 identities in a 29 nt sequence, or 23 identities in a 28 nt sequence, respectively. These mismatches were further identified in the regions between nucleotides 2-8 and 14-22 of the piRNAs, which have been previously described as the essential seed for the proper alignment between the piRNAs and their targets [39]. Moreover, the abundance of these piRNAs in the ovaries of control strain was only between three and nine copies.

## Discussion

In this study, we characterized the repertoire of nrEVEs integrated in the genomes of ten tephritid fruit flies with high impact on crop production. We retrieved an average of five nrEVEs in their genomes (range from 1 to 8), except for *E. diana* which presented a total of 22 nrEVEs. The high number of nrEVEs in *E. diana* did not correlate with the size of its reference genome, which was the second smallest among the species analysed (Table S2). Overall, the number of nrEVEs characterized in the genomes of tephritid fruit flies aligns with the number of nrEVEs reported in other dipteran families, with the notable exception of *Aedes* mosquitos. For instance, previous studies have found zero or one nrEVEs in the genome of the insect model *Drosophila melanogaster* [23, 40]*.* Other examples of the limited number of nrEVEs within the dipteran order are found for the biting midge *Culicoides sonorensis,* in which four nrEVEs were identified; the housefly *Musca domestica,* whose genome contains seven nrEVEs; and the sand fly *Phlebotomus papatasi*, which possesses a single nrEVE [23, 40]. In mosquitos, big differences have been revealed in the number of nrEVEs among Culicidae and *Anopheles* mosquitos*,* with an average of one and three nrEVEs per species; and *Aedes* mosquitos, in which more than 100 nrEVEs were initially identified [22, 41]. Subsequent studies have expanded the nrEVEs repertoire in *Aedes* mosquitoes to up to 200 [23–25, 42], reflecting that the quality of the reference genome assemblies, the selection of the viral query, and the filtering parameters are key for the identification of nrEVEs. In this vein, it is possible that the number of the nrEVEs retrieved in tephritid fruit flies will increase in the future as genome assembly quality improves and new viral species infecting insects will be discovered [29, 30, 28].

In concordance with previous studies, our findings support the notion that nrEVEs are predominantly derived from insertions of negative ssRNA viruses [23, 40]. Among these, *Rhabdoviridae-*derived nrEVEs were the most abundant, as previously observed in mosquitos [22]. Interestingly, this pattern extends beyond the phylum Arthropoda, since negative ssRNA viruses from the *Bornaviridae* and *Filoviridae* families represented the 72% of the nrEVEs discovered in the genomes of thirteen Australian marsupial species [43]. Our study also confirms the prevalence of structural gene-coding regions in nrEVEs, as previously reported in other insect species [22, 23]. In particular, 61% of the nrEVEs retrieved in tephritid fruit flies were derived from viral nucleocapsid and glycoprotein genes, suggesting that the transcripts encoding these proteins are more prone to insertion than other viral transcripts. Notably, this observation cannot be solely attributed to the expression levels of viral mRNAs since nucleoprotein genes are typically located at 5’end and transcription initiation occurs at the 3’end in negative single-stranded RNA (ssRNA) [44]. Instead, it is tempting to hypothesize that nucleocapsid and glycoprotein sequences were selectively maintained in true fruit fly genomes because they cause a benefit for the host fitness, although more research need to be done to test this hypothesis.

nrEVEs reflect the long-term and intimate relationships of viruses with their hosts, and their identification can shed light into past and present host distribution of viral genera and species. Our findings suggest two distinct scenarios for the origin of nrEVEs. Some nrEVEs may be orthologs, indicating they originated from viral insertions that occurred in the common ancestor of certain host lineages. On the contrary, other nrEVEs likely derive from integrations events occurred after speciation. To illustrate, we can date the origin of certain nrEVEs to the split of two species. For example, *B. dorsalis*, *B. tryoni* and *B. latifrons* diverged approximately 1.5 million of year ago [45], indicating that the non-ortholog nrEVEs identified in these three species represent relatively recent integrations. Interestingly, potential orthologous nrEVEs derived only from *Partitiviridae* and *Rhabdoviridae* families while known viruses capable of infecting multiple true fruit fly species mainly belong to the *Dicistroviridae* and *Iflaviridae* families. This lack of overlap between known viruses and nrEVEs points to a limited role of nrEVEs in providing an antiviral function. In line with this, we found no connections between known currently circulating viruses infecting a single tephritid species and the nrEVEs harboured in the correspondent genome. The only observed match was between the *nrEVE_CcapRhabdo_1* and the Ceratitis capitata sigmavirus 1, which shared a 66% nucleotide-level identity. All these findings support the hypothesis that the nrEVEs present in true fruit fly genomes are likely viral remnants of ancient viruses that once infected tephritids but do not confer current protection against viral infection. We suggest that viruses responsible for the existing nrEVEs have undergone significant mutations over time or even disappeared entirely, leaving behind traces in the form of the nrEVEs. However, we cannot rule out the possibility that additional viruses currently infecting tephritid fruit flies remain undiscovered.

Numerous studies have highlighted the important roles that nrEVEs can play in host antiviral immunity, both in vertebrates and invertebrates [18, 46–48]. In tephritid, many of identified nrEVEs are not transcribed. However, a subset of them undergoes active transcription within tissues, resulting in the production of transcripts that may represent either mRNAs or long non-coding RNAs. In the case of *C. capitata* and *Bactrocera* species, ten out of 28 nrEVEs were found to be actively transcribed. Among these, three contained a putative ORF and are therefore presumed to encode a fragment of viral mRNAs with structural functions. According to previous studies, the synthesis of viral proteins mediated by the nrEVEs may have a role on nrEVE-derived immunity. For instance, it has been hypothesized that nrEVE-derived viral proteins may act as antibodies that maintain an immune memory against circulating viruses in mammals [47]. On the other hand, nrEVEs may participate on the immune response by producing regulatory noncoding RNAs. Small RNAs produced by nrEVEs have been shown to limit the cognate virus replication in *Aedes* mosquitoes [18]. Additionally, a recombinant Sindbis virus modified to contain a sequence complementary to an *A. aegypti* nrEVE failed to replicate in Aag2 cells [17]. Our results indicate that two *C. capitata* nrEVEs may be a source of piRNAs in ovaries, and one of them also in somatic tissues. Both piRNA-producing nrEVEs were derived from a rhabdovirus and one of them, *nrEVE_CcapRhabdo_1,* was consistently transcribed in different *C. capitata* populations. However, when comparing the piRNAs produced by these nrEVEs with the known rhabdovirus specific to this species, Ceratitis capitata sigmavirus [49], no matches were found despite *nrEVE_CcapRhabdo_1* and Ceratitis capitata sigmavirus sharing 66% identity at the nucleotide level. This suggests that the piRNA pathway may not be involved in the immune defence against this particular virus.

In conclusion, our study confirms the integration of nrEVEs in the genomes of tephritid fruit fly species, as observed for other dipteran species, and unravels relatively recent insertion events occurred after the speciation of the extant tephritid lineages. The highly dynamic landscape of observed nrEVEs suggest that the nrEVEs may not be under selection. Despite some nrEVEs are transcriptionally active and produce piRNAs in *C. capitata*, the low sequence similarity observed between nrEVEs, piRNAs and known viruses indicates that nrEVEs from this species may not play a significant role in combating circulating viral infections. In the context of insect mass-rearing, an antiviral function of nrEVEs could have been exploited through a higher production of nrEVEs transcripts, although our results do not support this hypothesis in the case of true fruit flies. Overall, we provided valuable insights into the landscape of nrEVEs in true fruit flies, offering a comprehensive understanding of their presence and potential roles in fruit fly species.

## Supplementary information

ESM 1 (DOCX 291 kb)

## Data Availability

Small RNA sequencing data is available at the repositories of the National Centre of Biotechnology Information (NCBI).

## References

[1] Ismay JW (1992) Fruit flies of economic significance: their identification and bionomics. Bull Entomol Res 82:433–433. 10.1017/S0007485300041237

[2] Robinson AS (2002) Genetic sexing strains in medfly, *Ceratitis capitata*, sterile insect technique programmes. Genetica 116:5–13. 10.1023/a:1020951407069 12484522 10.1023/a:1020951407069

[3] Enkerlin WR (2005) Impact of fruit fly control programmes using the Sterile Insect Technique. Sterile Insect Tech:651–676. 10.1007/1-4020-4051-2_25

[4] Hendrichs J, Franz G, Rendon P (1995) Increased effectiveness and applicability of the sterile insect technique through male-only releases for control of Mediterranean fruit flies during fruiting seasons. J Appl Entomol 119. 10.1111/j.1439-0418.1995.tb01303.x

[5] Meats AW, Duthie R, Clift AD, Dominiak BC (2003) Trials on variants of the Sterile Insect Technique (SIT) for suppression of populations of the Queensland fruit fly in small towns neighbouring a quarantine (exclusion) zone. Aust J Exp Agric 43:389–395. 10.1071/EA02064

[6] Eilenberg J, Vlak JM, Nielsen-LeRoux C et al (2015) Diseases in insects produced for food and feed. J Insects as Food Feed 1:87–102. 10.3920/JIFF2014.0022

[7] Han Y, van Oers MM, van Houte S, Ros VID (2015) In: Mehlhorn H (ed) Virus-induced behavioural changes in insects BT - Host manipulations by parasites and viruses. Springer International Publishing, Cham, pp 149–174. 10.1007/978-3-319-22936-2_10

[8] Cory JS, Myers JH (2003) The ecology and evolution of insect baculoviruses. Annu Rev Ecol Evol Syst 34:239–272. 10.1146/annurev.ecolsys.34.011802.132402

[9] Zhang W, Gu Q, Niu J, Wang JJ (2020) The RNA virome and its dynamics in an invasive fruit fly, *Bactrocera dorsalis*, imply interactions between host and viruses. Microb Ecol 80:423–434. 10.1007/s00248-020-01506-9 32300830 10.1007/s00248-020-01506-9

[10] Sharpe SR, Morrow JL, Brettell LE et al (2021) Tephritid fruit flies have a large diversity of co-occurring RNA viruses. J Invertebr Pathol:107569. 10.1016/j.jip.2021.107569 10.1016/j.jip.2021.107569 33727045

[11] Hernández-Pelegrín L, Llopis-Giménez Á, Crava CM et al (2022) Expanding the medfly virome: viral diversity, prevalence, and sRNA profiling in mass-reared and field-derived medflies. Viruses 14. 10.3390/v14030623 10.3390/v14030623 PMC8955247 35337030

[12] Zhang W, Zhang Y-C, Wang Z-G et al (2022) The diversity of viral Community in invasive fruit flies (*Bactrocera and Zeugodacus*) revealed by meta-transcriptomics. Microb Ecol 83:739–752. 10.1007/s00248-021-01790-z 34173031 10.1007/s00248-021-01790-z

[13] Hernández-Pelegrín L, García-Martínez R, Llácer E et al (2023) Covert infection with an RNA virus affects medfly fitness and the interaction with its natural parasitoid *Aganaspis daci*. J Pest Sci 2004. 10.1007/s10340-023-01617-5

[14] Llopis-Giménez A, Maria González R, Millán-Leiva A et al (2017) Novel RNA viruses producing simultaneous covert infections in *Ceratitis capitata*. Correlations between viral titers and host fitness, and implications for SIT programs. J Invertebr Pathol 143:50–60. 10.1016/j.jip.2016.11.014 27914927 10.1016/j.jip.2016.11.014

[15] Bonning BC, Saleh M-C (2021) The interplay between viruses and RNAi pathways in insects. Annu Rev Entomol 66:61–79. 10.1146/annurev-ento-033020-090410 33417818 10.1146/annurev-ento-033020-090410

[16] Blair CD (2019) Deducing the role of virus genome-derived PIWI-associated RNAs in the mosquito-arbovirus arms race. Front Genet 10. 10.3389/fgene.2019.01114 10.3389/fgene.2019.01114 PMC6901949 31850054

[17] Tassetto M, Kunitomi M, Whitfield Z et al (2019) Control of RNA viruses in mosquito cells through the acquisition of vDNA and endogenous viral elements. Elife 8. 10.7554/eLife.41244 10.7554/eLife.41244 PMC6797480 31621580

[18] Suzuki Y, Baidaliuk A, Miesen P et al (2020) Non-retroviral endogenous viral element limits cognate virus replication in *Aedes aegypti* ovaries. Curr Biol 30:3495–3506. 10.1016/j.cub.2020.06.057 32679098 10.1016/j.cub.2020.06.057 PMC7522710

[19] Miesen P, Joosten J, van Rij RP (2016) PIWIs Go Viral: arbovirus-derived piRNAs in vector mosquitoes. PLoS Pathog 12. 10.1371/journal.ppat.1006017 10.1371/journal.ppat.1006017 PMC5198996 28033427

[20] Brennecke J, Aravin AA, Stark A et al (2007) Discrete small RNA-generating loci as master regulators of transposon activity in Droso. Cell 128:1089–1103. 10.1016/j.cell.2007.01.043 17346786 10.1016/j.cell.2007.01.043

[21] Luo Z, Ren H, Mousa JJ et al (2017) The PacC transcription factor regulates secondary metabolite production and stress response, but has only minor effects on virulence in the insect pathogenic fungus *Beauveria bassiana*. Environ Microbiol 19:788–802. 10.1111/1462-2920.13648 28083986 10.1111/1462-2920.13648

[22] Palatini U, Miesen P, Carballar-Lejarazu R et al (2017) Comparative genomics shows that viral integrations are abundant and express piRNAs in the arboviral vectors *Aedes aegypti and Aedes albopictus*. BMC Genom 18:512. 10.1186/s12864-017-3903-3 10.1186/s12864-017-3903-3 PMC5497376 28676109

[23] Russo AG, Kelly AG, Enosi Tuipulotu D et al (2019) Novel insights into endogenous RNA viral elements in *Ixodes scapularis* and other arbovirus vector genomes. Virus Evol 5. 10.1093/ve/vez010 10.1093/ve/vez010 PMC6580184 31249694

[24] Whitfield ZJ, Dolan PT, Kunitomi M et al (2017) The diversity, structure, and function of heritable adaptive immunity sequences in the *Aedes aegypti* genome. Curr Biol 27:3511–3519.e7. 10.1016/j.cub.2017.09.067 29129531 10.1016/j.cub.2017.09.067 PMC5698160

[25] Crava CM, Varghese FS, Pischedda E et al (2021) Population genomics in the arboviral vector *Aedes aegypti* reveals the genomic architecture and evolution of endogenous viral elements. Mol Ecol 30:1594–1611. 10.1111/mec.15798 33432714 10.1111/mec.15798 PMC8048955

[26] Johnson WE (2015) Endogenous retroviruses in the genomics era. Annu Rev Virol 2. 10.1146/annurev-virology-100114-054945 10.1146/annurev-virology-100114-054945 26958910

[27] Aswad A, Katzourakis A (2016) Paleovirology: the study of endogenous viral elements. Caister Academic press. 10.21775/9781910190234

[28] Shi M, Lin XD, Tian JH et al (2016) Redefining the invertebrate RNA virosphere. Nature 540:539–543. 10.1038/nature20167 27880757 10.1038/nature20167

[29] Haoming W, Rui P, Tong C et al (2021) Abundant and diverse RNA viruses in insects revealed by RNA-Seq analysis: ecological and evolutionary implications. mSystems 5:e00039-20. 10.1128/mSystems.00039-20 10.1128/mSystems.00039-20 PMC7343303 32636338

[30] Käfer S, Paraskevopoulou S, Zirkel F et al (2019) Re-assessing the diversity of negative strand RNA viruses in insects. PLoS Pathog 15:e1008224. 10.1371/journal.ppat.1008224 31830128 10.1371/journal.ppat.1008224 PMC6932829

[31] Bolger AM, Lohse M, Usadel B (2014) Trimmomatic: a flexible trimmer for Illumina sequence data. Bioinformatics 30:2114–2120. 10.1093/bioinformatics/btu170 24695404 10.1093/bioinformatics/btu170 PMC4103590

[32] Grabherr MG, Haas BJ, Yassour M et al (2011) Full-length transcriptome assembly from RNA-Seq data without a reference genome. Nat Biotechnol 29:644–652. 10.1038/nbt.1883 21572440 10.1038/nbt.1883 PMC3571712

[33] Langmead B, Salzberg SL (2012) Fast gapped-read alignment with Bowtie 2. Nat Methods 9:357–360. 10.1038/nmeth.1923 22388286 10.1038/nmeth.1923 PMC3322381

[34] Li B, Dewey CN (2011) RSEM: Accurate transcript quantification from RNA-Seq data with or without a reference genome. BMC Bioinform 12. 10.1186/1471-2105-12-323 10.1186/1471-2105-12-323 PMC3163565 21816040

[35] Warnes GR, Bolker B, Bonebakker L et al (2009) gplots: various R programming tools for plotting data. R Packag version 2:1. 10.1186/1471-2105-12-323

[36] Martin M (2011) Cutadapt removes adapter sequences from high-throughput sequencing reads. EMBnet.journal; Vol 17, No 1 Next Gener Seq Data Anal. 10.14806/ej.17.1.200

[37] Robinson JT, Thorvaldsdóttir H, Winckler W et al (2011) Integrative genomics viewer. Nat Biotechnol 29. 10.1038/nbt.1754 10.1038/nbt.1754 PMC3346182 21221095

[38] Lewis SH, Quarles KA, Yang Y et al (2018) Pan-arthropod analysis reveals somatic piRNAs as an ancestral defence against transposable elements. Nat Ecol Evol 2:174–181. 10.1038/s41559-017-0403-4 29203920 10.1038/s41559-017-0403-4 PMC5732027

[39] Zhang D, Tu S, Stubna M et al (2018) The piRNA targeting rules and the resistance to piRNA silencing in endogenous genes. Science 359:587–592. 10.1126/science.aao2840 29420292 10.1126/science.aao2840 PMC5939965

[40] Ter Horst AM, Nigg JC, Dekker FM, Falk BW (2019) Endogenous viral elements are widespread in arthropod genomes and commonly give rise to PIWI-interacting RNAs. J Virol 93. 10.1128/JVI.02124-18 10.1128/JVI.02124-18 PMC6401445 30567990

[41] Lequime S, Lambrechts L (2017) Discovery of flavivirus-derived endogenous viral elements in *Anopheles* mosquito genomes supports the existence of Anopheles-associated insect-specific flaviviruses. Virus Evol 3. 10.1093/ve/vew035 10.1093/ve/vew035 PMC5217911 28078104

[42] Pischedda E, Palatini U, Crava MC et al (2019) Discovery of novel endogenous viral elements in *Aedes spp*. mosquitoes. Access Microbiol 1. 10.1099/acmi.imav2019.po0014

[43] Harding EF, Russo AG, Yan GJH et al (2021) Ancient viral integrations in marsupials: a potential antiviral defence. Virus Evol 7. 10.1093/ve/veab076 10.1093/ve/veab076 PMC8449507 34548931

[44] Payne S (2017) Introduction to RNA viruses. Viruses:97–105. 10.1016/B978-0-12-803109-4.00010-6

[45] Valerio F, Zadra N, Rota-Stabelli O, Ometto L (2022) The impact of fast radiation on the phylogeny of *Bactrocera* fruit flies as revealed by multiple evolutionary models and mutation rate-calibrated clock. Insects 13. 10.3390/insects13070603 10.3390/insects13070603 PMC9319077 35886779

[46] Blair CD, Olson KE, Bonizzoni M (2020) The widespread occurrence and potential biological roles of endogenous viral elements in insect genomes. Curr Issues Mol Biol 34:13–30. 10.21775/cimb.034.013 31167954 10.21775/cimb.034.013

[47] Skirmuntt EC, Escalera-Zamudio M, Teeling EC et al (2020) The potential role of endogenous viral elements in the evolution of bats as reservoirs for zoonotic viruses. Annu Rev Virol 7. 10.1146/annurev-virology-092818-015613 10.1146/annurev-virology-092818-015613 32432980

[48] Ophinni Y, Palatini U, Hayashi Y, Parrish NF (2019) piRNA-guided CRISPR-like immunity in eukaryotes. Trends Immunol 40:998–1010. 10.1016/j.it.2019.09.003 31679813 10.1016/j.it.2019.09.003

[49] Longdon B, Day JP, Schulz N et al (2017) Vertically transmitted rhabdoviruses are found across three insect families and have dynamic interactions with their hosts. Proc R Soc B Biol Sci 284. 10.1098/rspb.2016.2381 10.1098/rspb.2016.2381 PMC5310039 28100819

