# Supplementary material for: Non-retroviral Endogenous Viral Elements in Tephritid Fruit Flies Reveal Former Viral Infections Not Related to Known Circulating Viruses
Source: Microb Ecol. 2023 Dec 1;87(1):7. doi: 10.1007/s00248-023-02310-x (PMC10689555; doi:10.1007/s00248-023-02310-x)
Supplement: Supplementary file 1 — (DOCX 291 kb) [file 248_2023_2310_MOESM1_ESM.docx]

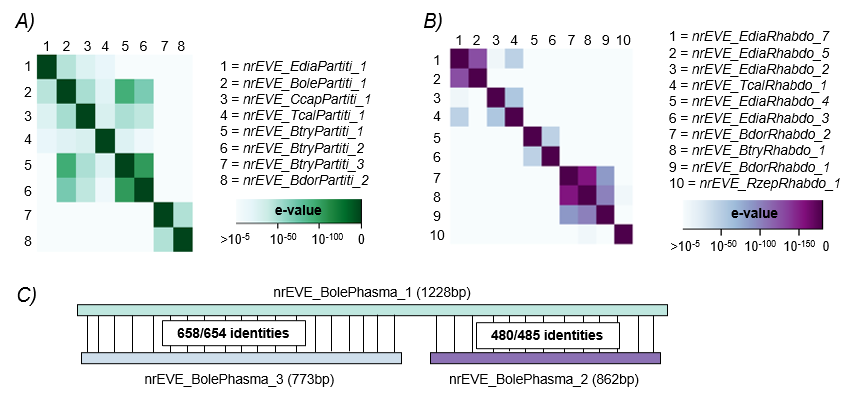


**Figure S1.** Homology at the nucleotide level between the nrEVEs classified within the A) Partitiviridae, B) Rhabdoviridae and C) Phasmaviridae families. For the first two, results were visualized as a HeatMap containing the e-value of the pairwise sequence comparisons. Additional nrEVEs derived from the *Partitiviridae* or *Rhabdoviridae* families which presented a similarity below the threshold (e-value > 10^-5^) were not displayed in the graph. For the three nrEVEs derived from the *Phasmaviridae* family, results were shown as a representation of the mapping, with the indication of the number of identities between the sequences.


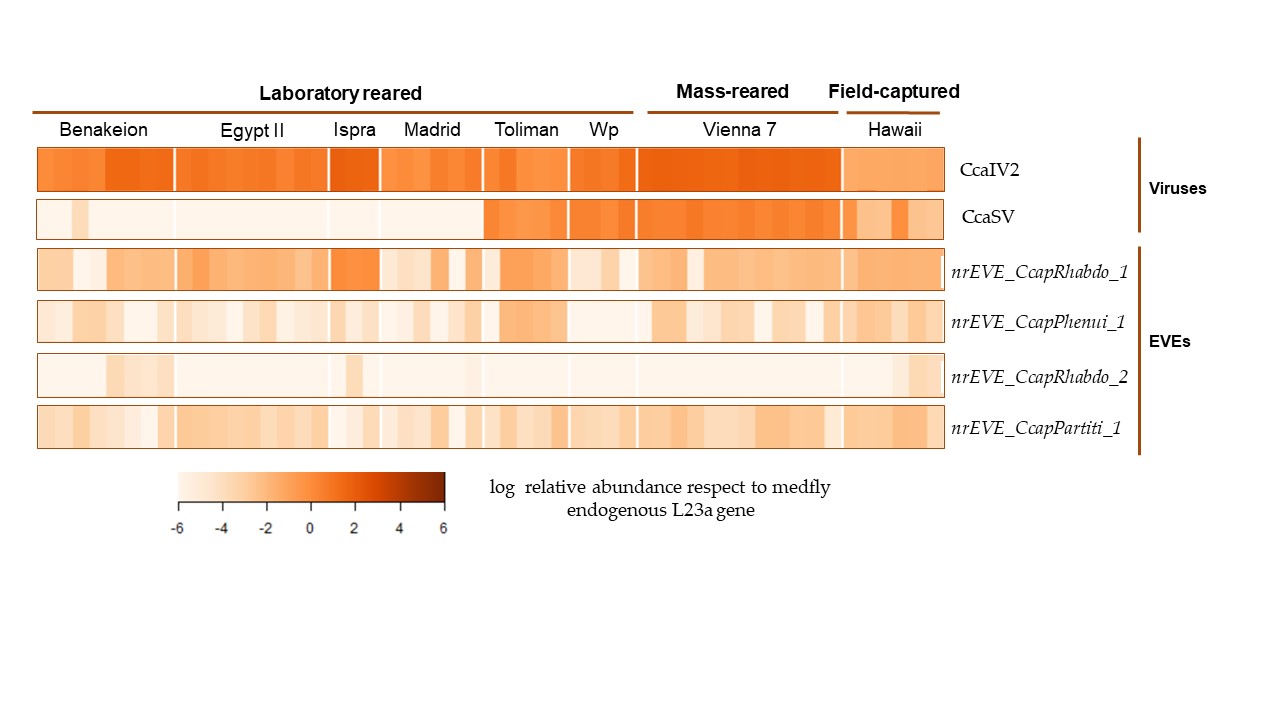


**Figure S2.** Expression of the 4 nrEVEs described in *C. capitata* in 53 transcriptomes of the species obtained from the Sequence Read Archive (SRA) on NCBI. Additionally, the expression of two viruses of the species was assessed. Ceratitis capitata Iflavirus 2 expression was obtained as positive control since it has been shown that CcaIV2 is ubiquitously distributed in *C. capitata.* Ceratitis capitata Sigmavirus (CcaSV) expression was calculated due to the sequence similarity between this virus and *nrEVE_Rhabdo_1.*


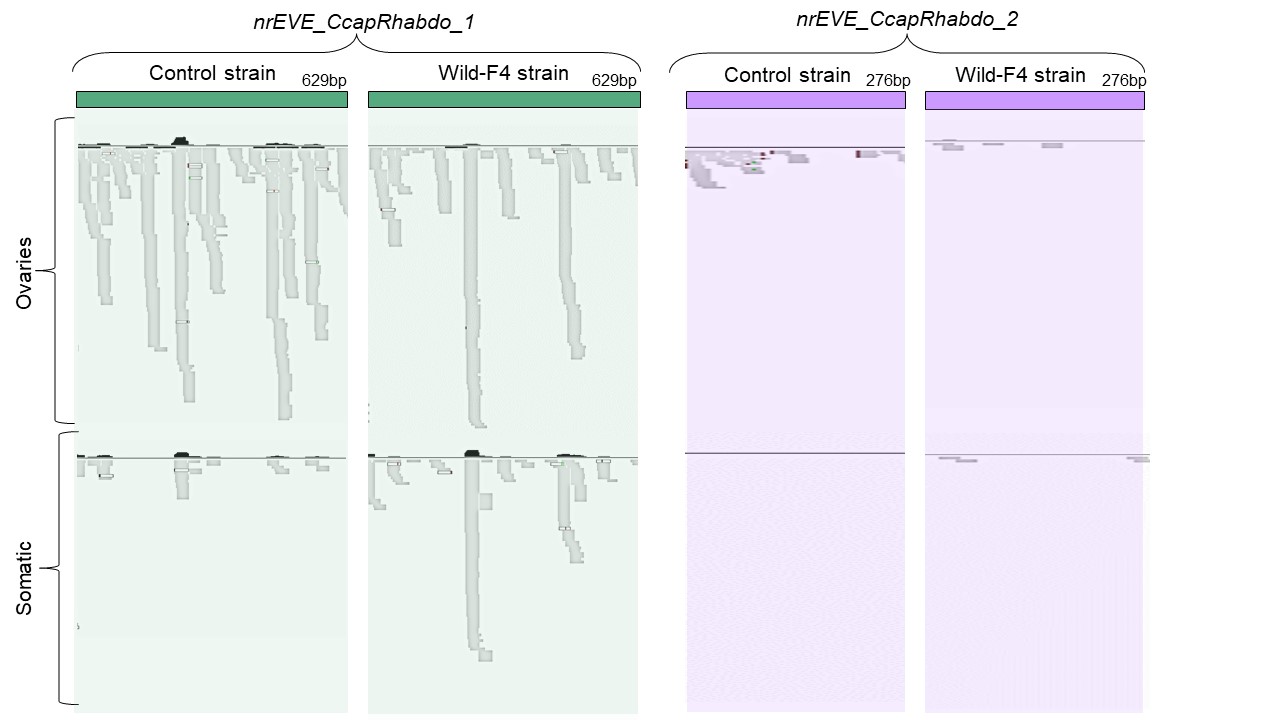


**Figure S3.** Mapping profile of the small RNAs across the sequence of the nrEVEs identified in the genome of C. capitata: *nrEVE_CcapRhabdo_1* and *nrEVE_CcapRhabdo_2*.

**Table S1.** Viral genomes used to test the similarity between nrEVEs and circulating viruses. The last column represents the groups of RNA viruses which share more than 95% similarities at the nucleotide level after blastn. A proposed common name has been added for each group of viruses fulfilling the 95% similarity criteria.

| **Virus** | **GenBank** | **Taxon** | **>95% nucleotide similarity with:** | **Proposed common name** |
| --- | --- | --- | --- | --- |
| Bactrocera bryoniae dicistrovirus 1 | HG994131.1 | [2814032](https://www.ncbi.nlm.nih.gov/Taxonomy/Browser/wwwtax.cgi?id=2814032) | Bactrocera frauenfeldi dicistrovirus 1  Bactrocera kraussi dicistrovirus 1  Bactrocera musae dicistrovirus 1 | Bactrocera species dicistrovirus 1 |
| Bactrocera bryoniae dicistrovirus 2 | HG994132.1 | [2814033](https://www.ncbi.nlm.nih.gov/Taxonomy/Browser/wwwtax.cgi?id=2814033) | Bactrocera frauenfeldi dicistrovirus 2  Bactrocera musae dicistrovirus 2 | Bactrocera species dicistrovirus 2 |
| Bactrocera bryoniae iflavirus 1 | HG993808.1 | [2812444](https://www.ncbi.nlm.nih.gov/Taxonomy/Browser/wwwtax.cgi?id=2812444) | Bactrocera tryoni iflavirus 1  Bactrocera tryoni iflavirus 2 | Bactrocera species  iflavirus 1 |
| Bactrocera dorsalis borna-like virus | MN745081.1 | [2760893](https://www.ncbi.nlm.nih.gov/Taxonomy/Browser/wwwtax.cgi?id=2760893) | Bactrocera dorsalis xinmovirus 2 | Bactrocera dorsalis xinmovirus 2 |
| Bactrocera dorsalis cripavirus | MN738553.1 | [2739222](https://www.ncbi.nlm.nih.gov/Taxonomy/Browser/wwwtax.cgi?id=2739222) | - | - |
| Bactrocera dorsalis negev-like virus 1 | MW310386.1 | [2933669](https://www.ncbi.nlm.nih.gov/Taxonomy/Browser/wwwtax.cgi?id=2933669) | Bactrocera dorsalis negev-like virus 2  Bactrocera dorsalis negev-like virus 3 | Bactrocera dorsalis negev-live virus 1 |
| Bactrocera dorsalis negev-like virus 2 | HG993812.1 | [2812452](https://www.ncbi.nlm.nih.gov/Taxonomy/Browser/wwwtax.cgi?id=2812452) | Bactrocera dorsalis negev-like virus 1  Bactrocera dorsalis negev-like virus 3 | Bactrocera dorsalis negev-live virus 1 |
| Bactrocera dorsalis negev-like virus 3 | HG993813.1 | [2812453](https://www.ncbi.nlm.nih.gov/Taxonomy/Browser/wwwtax.cgi?id=2812453) | Bactrocera dorsalis negev-like virus 1  Bactrocera dorsalis negev-like virus 2 | Bactrocera dorsalis negev-live virus 1 |
| Bactrocera dorsalis nora virus | MN738554.1 | [2739231](https://www.ncbi.nlm.nih.gov/Taxonomy/Browser/wwwtax.cgi?id=2739231) |  | - |
| Bactrocera dorsalis picorna-like virus 1 | MW310352.1 | [2933678](https://www.ncbi.nlm.nih.gov/Taxonomy/Browser/wwwtax.cgi?id=2933678) | Bactrocera dorsalis picorna-like virus 2 | Bactrocera dorsalis  picorna-like virus 1 |
| Bactrocera dorsalis picorna-like virus 2 | HG994136.1 | [2814078](https://www.ncbi.nlm.nih.gov/Taxonomy/Browser/wwwtax.cgi?id=2814078) | Bactrocera dorsalis picorna-like virus 1 | Bactrocera dorsalis  picorna-like virus 1 |
| Bactrocera dorsalis sigmavirus | MN745080.1 | [2760896](https://www.ncbi.nlm.nih.gov/Taxonomy/Browser/wwwtax.cgi?id=2760896) | - | - |
| Bactrocera dorsalis toti-like virus 1 | MN745082.1 | [2760897](https://www.ncbi.nlm.nih.gov/Taxonomy/Browser/wwwtax.cgi?id=2760897) | - | - |
| Bactrocera dorsalis toti-like virus 2 | MN745083.1 | [2760898](https://www.ncbi.nlm.nih.gov/Taxonomy/Browser/wwwtax.cgi?id=2760898) | - | - |
| Bactrocera dorsalis xinmovirus 2 | HG994135.1 | [2814077](https://www.ncbi.nlm.nih.gov/Taxonomy/Browser/wwwtax.cgi?id=2814077) | Bactrocera dorsalis borna-like virus | Bactrocera dorsalis xinmovirus 2 |
| Bactrocera frauenfeldi dicistrovirus 1 | HG994130.1 | [2814031](https://www.ncbi.nlm.nih.gov/Taxonomy/Browser/wwwtax.cgi?id=2814031) | Bactrocera bryoniae dicistrovirus 1  Bactrocera kraussi dicistrovirus 1  Bactrocera musae dicistrovirus 1 | Bactrocera species dicistrovirus 1 |
| Bactrocera frauenfeldi dicistrovirus 2 | HG993809.1 | [2812449](https://www.ncbi.nlm.nih.gov/Taxonomy/Browser/wwwtax.cgi?id=2812449) | Bactrocera bryoniae dicistrovirus 2  Bactrocera musae dicistrovirus 2 | Bactrocera species dicistrovirus 2 |
| Bactrocera jarvisi toti-like virus 1 | HG994127.1 | [2814076](https://www.ncbi.nlm.nih.gov/Taxonomy/Browser/wwwtax.cgi?id=2814076) | Bactrocera tryoni toti-like virus 1 | Bactrocera species  toti-like virus 1 |
| Bactrocera kraussi dicistrovirus 1 | HG994128.1 | [2814036](https://www.ncbi.nlm.nih.gov/Taxonomy/Browser/wwwtax.cgi?id=2814036) | Bactrocera frauenfeldi dicistrovirus 1  Bactrocera bryoniae dicistrovirus 1  Bactrocera musae dicistrovirus 1 | Bactrocera species dicistrovirus 1 |
| Bactrocera kraussi iflavirus 1 | HG993805.1 | [2812441](https://www.ncbi.nlm.nih.gov/Taxonomy/Browser/wwwtax.cgi?id=2812441) | Bactrocera kraussi iflavirus 2  Bactrocera kraussi iflavirus 3 | Bactrocera kraussi  iflavirus 1 |
| Bactrocera kraussi iflavirus 2 | HG993806.1 | [2812442](https://www.ncbi.nlm.nih.gov/Taxonomy/Browser/wwwtax.cgi?id=2812442) | Bactrocera kraussi iflavirus 1  Bactrocera kraussi iflavirus 3 | Bactrocera kraussi  iflavirus 1 |
| Bactrocera kraussi iflavirus 3 | HG993807.1 | [2812443](https://www.ncbi.nlm.nih.gov/Taxonomy/Browser/wwwtax.cgi?id=2812443) | Bactrocera kraussi iflavirus 1  Bactrocera kraussi iflavirus 2 | Bactrocera kraussi  iflavirus 1 |
| Bactrocera kraussi negev-like virus 1 | HG994129.1 | [2814080](https://www.ncbi.nlm.nih.gov/Taxonomy/Browser/wwwtax.cgi?id=2814080) | Bactrocera tryoni negev-like virus | Bactrocera species  negev-like virus 1 |
| Bactrocera musae dicistrovirus 1 | HG994133.1 | [2814034](https://www.ncbi.nlm.nih.gov/Taxonomy/Browser/wwwtax.cgi?id=2814034) | Bactrocera frauenfeldi dicistrovirus 1  Bactrocera bryoniae dicistrovirus 1  Bactrocera kraussi dicistrovirus 1 | Bactrocera species dicistrovirus 1 |
| Bactrocera musae dicistrovirus 2 | HG994134.1 | [2814035](https://www.ncbi.nlm.nih.gov/Taxonomy/Browser/wwwtax.cgi?id=2814035) | Bactrocera bryoniae dicistrovirus 2  Bactrocera frauenfeldi dicistrovirus 2 | Bactrocera species dicistrovirus 2 |
| Bactrocera tryoni dicistrovirus 1 | MW208808.1 | [2795007](https://www.ncbi.nlm.nih.gov/Taxonomy/Browser/wwwtax.cgi?id=2795007) | - | - |
| Bactrocera tryoni dicistrovirus 2 | MW208809.1 | [2795008](https://www.ncbi.nlm.nih.gov/Taxonomy/Browser/wwwtax.cgi?id=2795008) | - | - |
| Bactrocera tryoni dicistrovirus 3 | HG994979.1 | [2815578](https://www.ncbi.nlm.nih.gov/Taxonomy/Browser/wwwtax.cgi?id=2815578) | - | - |
| Bactrocera tryoni dicistrovirus 4 | HG993802.1 | [2812446](https://www.ncbi.nlm.nih.gov/Taxonomy/Browser/wwwtax.cgi?id=2812446) | - | - |
| Bactrocera tryoni iflavirus 1 | MW208810.1 | [2795009](https://www.ncbi.nlm.nih.gov/Taxonomy/Browser/wwwtax.cgi?id=2795009) | Bactrocera bryoniae iflavirus 1  Bactrocera tryoni iflavirus 2 | Bactrocera species  iflavirus 1 |
| Bactrocera tryoni iflavirus 2 | HG994125.1 | [2814058](https://www.ncbi.nlm.nih.gov/Taxonomy/Browser/wwwtax.cgi?id=2814058) | Bactrocera bryoniae iflavirus 1  Bactrocera tryoni iflavirus 1 | Bactrocera species  iflavirus 1 |
| Bactrocera tryoni iflavirus 3 | HG994126.1 | [2814059](https://www.ncbi.nlm.nih.gov/Taxonomy/Browser/wwwtax.cgi?id=2814059) |  | No similarities |
| Bactrocera tryoni negev-like virus | HG993804.1 | [2812448](https://www.ncbi.nlm.nih.gov/Taxonomy/Browser/wwwtax.cgi?id=2812448) | Bactrocera kraussi negev-like virus 1 | Bactrocera species negev-like virus 1 |
| Bactrocera tryoni picorna-like virus | MW208812.1 | [2795010](https://www.ncbi.nlm.nih.gov/Taxonomy/Browser/wwwtax.cgi?id=2795010) | - | - |
| Bactrocera tryoni rhabdovirus 1 | MW208811.1 | [2795014](https://www.ncbi.nlm.nih.gov/Taxonomy/Browser/wwwtax.cgi?id=2795014) | - | - |
| Bactrocera tryoni toti-like virus 1 | HG993803.1 | [2812447](https://www.ncbi.nlm.nih.gov/Taxonomy/Browser/wwwtax.cgi?id=2812447) | Bactrocera jarvisi toti-like virus 1 | Bactrocera species toti-like virus 1 |
| Ceratitis capitata iflavirus 1 | Undefined | | - | - |
| Ceratitis capitata iflavirus 2 | OL957305.1 | [2932852](https://www.ncbi.nlm.nih.gov/Taxonomy/Browser/wwwtax.cgi?id=2932852) | - | - |
| Ceratitis capitata iflavirus 3 | HG994137.1 | [2814060](https://www.ncbi.nlm.nih.gov/Taxonomy/Browser/wwwtax.cgi?id=2814060) | - | - |
| Ceratitis capitata iflavirus 4 | HG994138.1 | [2814061](https://www.ncbi.nlm.nih.gov/Taxonomy/Browser/wwwtax.cgi?id=2814061) | - | - |
| Ceratitis capitata narnavirus | OL957306.1 | [2932853](https://www.ncbi.nlm.nih.gov/Taxonomy/Browser/wwwtax.cgi?id=2932853) | - | - |
| Ceratitis capitata negev-like virus 1 | HG994139.1 | [2814079](https://www.ncbi.nlm.nih.gov/Taxonomy/Browser/wwwtax.cgi?id=2814079) | - | - |
| Ceratitis capitata negev-like virus 2 | OL957307.1 | [2932854](https://www.ncbi.nlm.nih.gov/Taxonomy/Browser/wwwtax.cgi?id=2932854) | - | - |
| Ceratitis capitata nodavirus | OL957308.1 | [2932855](https://www.ncbi.nlm.nih.gov/Taxonomy/Browser/wwwtax.cgi?id=2932855) | - | - |
| Ceratitis capitata nora virus | Undefined | | - | - |
| Ceratitis capitata reo-like virus 1 | OL957310.1 | [2932856](https://www.ncbi.nlm.nih.gov/Taxonomy/Browser/wwwtax.cgi?id=2932856) | - | - |
| Ceratitis capitata totivirus 1 | OL957313.1 | [2932857](https://www.ncbi.nlm.nih.gov/Taxonomy/Browser/wwwtax.cgi?id=2932857) | - | - |
| Ceratitis capitata sigmavirus | NC_076146.1 | [1802949](https://www.ncbi.nlm.nih.gov/Taxonomy/Browser/wwwtax.cgi?id=1802949) | - | - |
| Ceratitis capitata virga-like virus 1 | Undefined | | - | - |
| [Zeugodacus cucurbitae toti-like virus](https://www.ncbi.nlm.nih.gov/Taxonomy/Browser/wwwtax.cgi?id=2933690) | MW310353.1 | [2933690](https://www.ncbi.nlm.nih.gov/Taxonomy/Browser/wwwtax.cgi?id=2933690) | - | - |
| [Zeugodacus cucurbitae negev-like virus](https://www.ncbi.nlm.nih.gov/Taxonomy/Browser/wwwtax.cgi?id=2933687) | MW310350.1 | [2933687](https://www.ncbi.nlm.nih.gov/Taxonomy/Browser/wwwtax.cgi?id=2933687) | - | - |

**Table S2.** Reference genomes used for the analysis of endogenous virus elements in tephritid fruit flies.

| **Scientific name** | **RefSeq** | **Assembly** | **Size (Mb)** |  |
| --- | --- | --- | --- | --- |
| *Bactrocera cucurbitae* | GCF_000806345.1 | ASM80634v1 | 374.8 | Fruit |
| *Bactrocera dorsalis* | GCF_000789215.1 | ASM78921v2 | 415 | Fruit |
| *Bactrocera latifrons* | GCF_001853355.1 | ASM185335v1 | 462,5 | Pepper |
| *Bactrocera oleae* | GCF_001188975.3 | MU_Boleae_v2 | 484,9 | Olive |
| *Bactrocera tryoni* | GCA_000695345.1 | Assembly 2.2 of *Bactrocera tryoni* genome | 519 | Fruit |
| *Ceratitis capitata* | GCF_000347755.3 | Ccap_2.1 | 436,5 | Fruit |
| *Eutreta diana* | GCA_001015115.1 | ASM101511v1 | 233,1 | A. tridentata. |
| *Rhagoletis zephyria* | GCF_001687245.2 | *Rhagoletis_zephyria*_1.1 | 1100 | Cherry |
| *Tephritis californica* | GCA_001017515.1 | ASM101751v1 | 342,3 | Unc. |
| *Trupanea jonesi* | GCA_001014665.1 | ASM101466v1 | 97,3 | Unc. |

**Table S3**. Available SRA datasets of 6 tephritid fruit fly species used to assess the expression of the nrEVEs.

| SRA accession number | Experiment Accession | Organism Name | Instrument | Submitter |
| --- | --- | --- | --- | --- |
| [SRR1993650](https://trace.ncbi.nlm.nih.gov/Traces/sra/?run=SRR1993650) | SRX1007579 | *Bactrocera latifrons* | Illumina HiSeq 2000 | USDA-ARS Pacific Basin Agricultural Research Center (PBARC) |
| [SRR1993648](https://trace.ncbi.nlm.nih.gov/Traces/sra/?run=SRR1993648) | SRX1007578 |  | Illumina HiSeq 2000 | USDA-ARS Pacific Basin Agricultural Research Center (PBARC) |
| [SRR1993646](https://trace.ncbi.nlm.nih.gov/Traces/sra/?run=SRR1993646) | SRX1007576 |  | Illumina HiSeq 2000 | USDA-ARS Pacific Basin Agricultural Research Center (PBARC) |
| [SRR1993647](https://trace.ncbi.nlm.nih.gov/Traces/sra/?run=SRR1993647) | SRX1007577 |  | Illumina HiSeq 2000 | USDA-ARS Pacific Basin Agricultural Research Center (PBARC) |
| [ERR3219438](https://trace.ncbi.nlm.nih.gov/Traces/sra/?run=ERR3219438) | ERX3246919 | *Bactrocera tryoni* | NextSeq 500 | UNIVERSITY OF NEW SOUTH WALES |
| [SRR9302948](https://trace.ncbi.nlm.nih.gov/Traces/sra/?run=SRR9302948) | SRX6078240 |  | Illumina HiSeq 2000 | QUT |
| [SRR8662649](https://trace.ncbi.nlm.nih.gov/Traces/sra/?run=SRR8662649) | SRX5459372 |  | Illumina HiSeq 2000 | University of New South Wales |
| [SRR8662654](https://trace.ncbi.nlm.nih.gov/Traces/sra/?run=SRR8662654) | SRX5459367 |  | Illumina HiSeq 2000 | University of New South Wales |
| [SRR5559327](https://trace.ncbi.nlm.nih.gov/Traces/sra/?run=SRR5559327) | SRX2822449 | *Bactrocera oleae* | Illumina Genome Analyzer | Bachtrog Lab, UC Berkeley |
| [SRR5559328](https://trace.ncbi.nlm.nih.gov/Traces/sra/?run=SRR5559328) | SRX2822448 |  | Illumina Genome Analyzer | Bachtrog Lab, UC Berkeley |
| [SRR826666](https://trace.ncbi.nlm.nih.gov/Traces/sra/?run=SRR826666) | SRX265051 |  | Illumina Genome Analyzer | UC Berkeley |
| [SRR826665](https://trace.ncbi.nlm.nih.gov/Traces/sra/?run=SRR826665) | SRX265050 |  | Illumina Genome Analyzer | UC Berkeley |
| [SRR10058476](https://trace.ncbi.nlm.nih.gov/Traces/sra/?run=SRR10058476) | SRX6792367 | *Zeugodacus cucurbitae* | HiSeq X Ten | Southwest University, China |
| [SRR10058486](https://trace.ncbi.nlm.nih.gov/Traces/sra/?run=SRR10058486) | SRX6792357 |  | HiSeq X Ten | Southwest University, China |
| [SRR10058487](https://trace.ncbi.nlm.nih.gov/Traces/sra/?run=SRR10058487) | SRX6792356 |  | HiSeq X Ten | Southwest University, China |
| [SRR10058496](https://trace.ncbi.nlm.nih.gov/Traces/sra/?run=SRR10058496) | SRX6792347 |  | HiSeq X Ten | Southwest University, China |
| [SRR9945666](https://trace.ncbi.nlm.nih.gov/Traces/sra/?run=SRR9945666) | SRX6694156 | *Bactrocera dorsalis* | Illumina HiSeq 2500 | Key laboratory of Entomology and Pest Control Engi |
| [SRR9945677](https://trace.ncbi.nlm.nih.gov/Traces/sra/?run=SRR9945677) | SRX6694145 |  | Illumina HiSeq 2500 | Key laboratory of Entomology and Pest Control Engi |
| [SRR1032039](https://trace.ncbi.nlm.nih.gov/Traces/sra/?run=SRR1032039) | SRX378864 |  | Illumina HiSeq 2000 | College of Plant Protection, Southwest University, |
| [SRR526902](https://trace.ncbi.nlm.nih.gov/Traces/sra/?run=SRR526902) | SRX170663 |  | Illumina HiSeq 2000 | College of Plant Protection southwest university |
| SRR16562767 | [SRX12764798](https://www.ncbi.nlm.nih.gov/sra/SRX12764798%5baccn%5d) | *Ceratitis capitata* | Illumina HiSeq 2000 | University of Valencia |
| SRR915844 | [SRX312188](https://www.ncbi.nlm.nih.gov/sra/SRX312188%5baccn%5d) |  | Illumina HiSeq 2000 | USDA-ARS Pacific Basin Agricultural Research Center (PBARC) |
| SRR915848 | [SRX312191](https://www.ncbi.nlm.nih.gov/sra/SRX312191%5baccn%5d) |  | Illumina HiSeq 2000 | USDA-ARS Pacific Basin Agricultural Research Center (PBARC) |
| SRR836188 | [SRX272876](https://www.ncbi.nlm.nih.gov/sra/SRX272876%5baccn%5d) |  | Illumina HiSeq 2000 | Baylor College of Medicine (BCM) |

**Table S4**. SRA datasets of *Ceratitis capitata* selected to assess the expression of the nrEVEs.

| **NCBI accession number** | **Sequencing method** | ***C. capitata* origin** | **Library details** | | **SRA accession number** |
| --- | --- | --- | --- | --- | --- |
| SRP133427 | Illumina HiSeq 2000 | Benakeion | Sexed 4-8h embryos | Male | SRR6780761 |
|  |  |  |  |  | SRR6780762 |
|  |  |  |  | Female | SRR6780763 |
|  |  |  |  |  | SRR6780764 |
| ERP119522 | Illumina NovaSeq 6000; pair end |  | Pool of 3 larvae | Immobilized larvae | ERR4026378 |
|  |  |  |  |  | ERR4026379 |
|  |  |  |  | Jumping larvae | ERR4026375 |
|  |  |  |  |  | ERR4026376 |
| SRP073787 | Illumina HiSeq 2000, single | Egypt II | Gastrointestinal tract | Virgin female | SRR3436822 |
|  |  |  |  |  | SRR3436813 |
|  |  |  | Reproductive tract | Mated male | SRR3436821 |
|  |  |  |  | Mated male | SRR3436820 |
|  |  |  |  | Mated female | SRR3436819 |
|  |  |  |  | Mated female | SRR3436818 |
|  |  |  |  | Virgin male | SRR3436817 |
|  |  |  |  | Virgin male | SRR3436816 |
|  |  |  |  | Virgin female | SRR3436815 |
|  |  |  |  | Virgin female | SRR3436814 |
| SRP021914 | Illumina Genome Analyzer Iix; pair end | Ispra | Adult | Male | SRR836190 |
|  |  |  |  | Female | SRR836189 |
|  |  |  | Embryo |  | SRR836188 |
| ERP124511 | Illumina HiSeq 2000 paired end sequencing | Madrid | Whole adult,  Control strain | Replicate 1 | ERR4690327 |
|  |  |  |  | Replicate 2 | ERR4690326 |
|  |  |  |  | Replicate 3 | ERR4690325 |
| SRP343008 |  |  | Whole adult,  W-1Kλ strain | Replicate 1 | SRR16562767 |
|  |  |  |  | Replicate 2 | SRR16562768 |
|  |  |  |  | Replicate 3 | SRR16562769 |
| SRP075464 | Illumina Genome Analyzer II | Toliman | Female ovary | | SRR3554596 |
|  |  |  | Elongated spermatids | | SRR3554595 |
|  |  |  | Round spermatids | | SRR3554594 |
|  |  |  | Late spermatocytes | | SRR3554593 |
|  |  |  | Early spermatocytes | | SRR3554592 |
| ERP119522 | Illumina NovaSeq 6000; pair end | Wp | Pool of 3 larvae | Immobilized larvae | ERR4026384 |
|  |  |  |  |  | ERR4026385 |
|  |  |  |  | Jumping larvae | ERR4026381 |
|  |  |  |  |  | ERR4026382 |
| SRP026213 | Illumina HiSeq 2000 | **Vienna 7** | Non-irradiated | Pupae | SRR915844 |
|  |  |  |  |  | SRR915840 |
|  |  |  |  |  | SRR915839 |
|  |  |  |  | Adult | SRR915838 |
|  |  |  |  |  | SRR915837 |
|  |  |  |  |  | SRR915836 |
|  |  |  | Irradiated | Pupae | SRR915835 |
|  |  |  |  |  | SRR915834 |
|  |  |  |  |  | SRR915833 |
|  |  |  |  | Adult | SRR915832 |
|  |  |  |  |  | SRR915831 |
|  |  |  |  |  | SRR915830 |
| SRP026213 | Illumina HiSeq 2000 | **Hawaiian** | Whole body | Wild pupae | SRR915852 |
|  |  |  |  |  | SRR915851 |
|  |  |  |  |  | SRR915850 |
|  |  |  |  | Wild adults | SRR915848 |
|  |  |  |  |  | SRR915846 |
|  |  |  |  |  | SRR915845 |

**Table S5.** Medfly nrEVEs presence in whole genome sequencing datasets of different medfly strains and conditions.

| **Run accession** | **Strain** | **Experiment Title** | **strain** | **Geographic location** | **nrEVEs presence** |
| --- | --- | --- | --- | --- | --- |
| [ERR4026339](https://trace.ncbi.nlm.nih.gov/Traces/sra/?run=ERR4026339) | Vienna 7 (female) | Illumina HiSeq 4000 paired end sequencing | 4-4_V7_F | Vienna | 4/4 |
| [ERR4026338](https://trace.ncbi.nlm.nih.gov/Traces/sra/?run=ERR4026338) | Vienna 7 (male) | Illumina HiSeq 4000 paired end sequencing | 3-3_V7_M | Vienna | 4/4 |
| [ERR4026337](https://trace.ncbi.nlm.nih.gov/Traces/sra/?run=ERR4026337) | Egypt II (female) | Illumina HiSeq 4000 paired end sequencing | Sample_6-6_EGII_F | Egypt | 4/4 |
| [ERR4026336](https://trace.ncbi.nlm.nih.gov/Traces/sra/?run=ERR4026336) | Egypt II (male) | Illumina HiSeq 4000 paired end sequencing | Sample_5-5_EGII_M | Egypt | 4/4 |
| [SRR847379](https://trace.ncbi.nlm.nih.gov/Traces/sra/?run=SRR847379) | Ispra (female) | WGS sequencing of *Ceratitis capitata* female | *Ceratitis capitata*- inbred Ispra strain | Italy, Pavia | 4/4 |
| [SRR847380](https://trace.ncbi.nlm.nih.gov/Traces/sra/?run=SRR847380) | Ispra (male) | WGS sequencing of *Ceratitis capitata* pooled males | inbred Ispra strain from pooled male organism | Italy, Pavia | 4/4 |
| [SRR11649129](https://trace.ncbi.nlm.nih.gov/Traces/sra/?run=SRR11649129) | TSL , Hawaii (male) | DNAseq of *Ceratitis capitata* | F2 of CDFA TSL strain cross wild type HiMed, White male 3M | USA: Hilo, Hawaii | 4/4 |
| [SRR11649132](https://trace.ncbi.nlm.nih.gov/Traces/sra/?run=SRR11649132) | TSL , Hawaii (female) | DNAseq of *Ceratitis capitata* | F2 of CDFA TSL strain cross wild type HiMed, Brown Female 44F | USA: Hilo, Hawaii | 4/4 |
| [ERR171452](https://trace.ncbi.nlm.nih.gov/Traces/sra/?run=ERR171452) | Field (Italy: Sardania) |  | fresh specimen, head and thorax (2010-2012) | Italy: Sardania | 4/4 |
| [ERR171450](https://trace.ncbi.nlm.nih.gov/Traces/sra/?run=ERR171450) | Field (USA: Los Angeles) |  | archived specimen (1995-2004) thorax and head | USA: Los Angeles | 4/4 |
